# A Retrospective Analysis to Validate the Alarm Signs Used in the CEDAP-Plus Study

**DOI:** 10.5402/2011/271047

**Published:** 2011-06-16

**Authors:** Hou-De Zhang, Mu-Xian Lin, Qu Zhang

**Affiliations:** Department of Gastroenterology, Shenzhen Nanshan Hospital, Guangdong Medical College, Shenzhen, Guangdong 518052, China

## Abstract

*Background and Study Aim*. This study aimed to validate the alarm signs used in the 2007 German CEDAP-Plus study for indicating capsule endoscopy in patients who have idiopathic chronic abdominal pain. *Patients and Methods*. We retrospectively reviewed the cases of all patients who underwent capsule endoscopy at our institution between August 2007 and August 2009 for chronic hitherto undiagnosed abdominal pain, despite previous investigations. The demographic data, indications, findings, and diagnoses were recorded, as were the alarm signs (i.e., 10% loss of weight within 3 months, suspected small intestinal bleed or chronic anemia, and laboratory indications of inflammation). *Results*. Alarm signs were found in only 4 of the 62 included patients. Capsule endoscopy revealed findings that led to diagnoses of Crohn's disease (*n* = 4), tuberculosis (*n* = 1), gastrointestinal stromal tumors (*n* = 3), and hookworm (*n* = 1); these diagnoses included 100% (4/4) of the patients with alarm signs, but only 8.6% (5/58) of patients without them. However, 55.6% (5/9) of patients with clinically capsule endoscopy findings reported no alarm signs. *Conclusions*. Although selecting patients based on the alarm signs may increase the yield of capsule endoscopy, the alarm sign criteria appear to have low sensitivity.

## 1. Introduction

Capsule endoscopy has been unequivocally recommended as the first line test for the evaluation of obscure gastrointestinal bleed, suspected Crohn's disease, and polyposis syndromes, since excellent effectiveness on these entities has been confirmed by many retrospective and prospective studies. But consensus has not been achieved on the indication for this noninvasive diagnostic modality in the evaluation of chronic abdominal pain and diarrhea, which are among the most common reasons referral to gastroenterologists [1]. Some studies reported that capsule endoscopy was not much of value in patients whose symptom was chronic abdominal pain or diarrhea alone [2–4]. other studies found that capsule endoscopy was helpful if chronic abdominal pain or diarrhea was accompanied by additional alarm signs, such as weight loss, anemia, inflammation, malabsorption, or obstruction [5–7]. In the only published, prospective, multicenter trial to date, the CEDAP-Plus study, May et al. [7] reported that careful selection of patients based on the presence of one or more alarm signs might increase the diagnostic yield of capsule endoscopy to as high as 40%. Hence the aim of the current analysis was to validate the alarm signs used in CEDAP-Plus study.

## 2. Patients and Methods

We retrospectively reviewed the medical records of all 180 patients who had undergone diagnostic capsule endoscopy studies at Shenzhen Nanshan Hospital between August 1, 2007 and August 30, 2009. Data extracted from the medical records included age, sex, indication for procedure, detailed history, physical examination, laboratory results, and findings from previous endoscopic and radiologic tests and abdominal ultrasonography. From 180 patients in this group, we selected those with a history of undiagnosed chronic abdominal pain. The admission criteria were that the abdominal pain had lasted at least 3 months, and that the following investigations had demonstrated no abnormalities sufficient to yield a diagnosis: upper and lower gastrointestinal endoscopy, abdominal ultrasonography, and/or gastrointestinal radiology. 

The patients who met these criteria were then grouped according to whether or not they had reported any of the alarm signs defined in the CEDAP-Plus study. These included (1) weight loss of ≥10% within 3 months (2) chronic suspected mid-gastrointestinal bleeding and/or chronic iron-deficiency anemia for 3 months or more, with hemoglobin levels ≤11 g/dL for women and 13 g/dL for men and (3) pathology laboratory results suggesting inflammation, such as erythrocyte sedimentation rate (ESR) >15/30 mm/h, Westergren method; C-reactive protein (CRP) >2 ng/dL; thrombocytosis >400 000/mL, and/or leukocytosis >15 000/mL.

All patients gave informed consent prior to capsule endoscopy (OMOM Capsule Endoscope System, Jinshan, China). A polyethylene glycol (2 L) and simethicone preparation was administered the day before the procedure, and the video capsule ingested after an overnight fast. Patients were allowed to drink clear fluids 2 h after swallowing the capsule and to consume a light meal 4 h after ingestion. They returned the recording system approximately 8 h after having swallowed the video capsule. The data recorder was subsequently downloaded to a workstation and the video analyzed by a gastroenterologist proficient in the technique.

Intestinal preparation for capsule endoscopy was defined as excellent (no debris, completely visualization of the mucosa), good (some debris), fair (several areas with incomplete visualization), or poor (large amounts of debris that compromised results). 

Lesions found on capsule endoscopy were classified according to whether or not they could explain the patient's symptoms. Significant findings included multiple erosions or ulcerations, diffuse erythema and edema, mucosal atrophy, strictures, tumors, diffuse lymphangiectasia, an inflamed secondary diverticulum or Meckel's diverticulum, and intestinal worms. Lesions considered not significant included arteriovenous malformations, focal lymphangiectasia, hyperplastic follicles, small lipomas, solitary diverticuli, focal erythema, or scattered and small mucosal erosions or breaks.

Descriptive statistics were used to report the demographics and clinical characteristics of patients. We reported the prevalence of findings, the complete visualization, and incidence of retention of the capsule. Statistical uncertainty was quantified by calculating 95% confidence intervals (CIs) using the binomial method. Fisher's test was used to compare diagnostic yield difference between the patients with and without the alarm signs defined by the CEDAP-Plus study. Both the patients and the Hospital Ethics Committee agreed on our research.

## 3. Results

Over the 24-month study period, 62 patients were considered to have obscure chronic abdominal pain and were included in the study. Of these, 51 had chronic diarrhea, 6 had constipation, and 5 reported normal bowel movements. The median age was 43 years (range 20–78), and there were 27 males and 35 females (43.5 and 56.5%, resp.). Only 4 patients had alarm signs as defined by the CEDAP-Plus Study.

Visualization of the entire small bowel with cecum was achieved in 61 of the 62 patients (98.0%, 95% CI = 91.4–100%). Preparation for capsule endoscopy was excellent in 40 patients (64.5%), good in 21 (33.8%), and fair in 1 (1.6%).

Of the 62 patients, capsule endoscopy revealed clinically significant findings that were able to explain the patient's symptoms in 9 cases (diagnostic yield, 14.5%; 95% CI = 6.9–25.8%). The final diagnoses of these patients included Crohn's disease in 4 patients, tuberculous peritonitis in 1, gastrointestinal stromal tumors (GISTs) in 3, and hookworm in 1. A total of 16 patients (25.8%, 95% CI = 11.5–38.5%) had lesions not related to their symptoms, including arteriovenous malformation in 3 patients, focal lymphangiectasia in 4, hyperplasic follicle in 5, solitary diverticulum in 1, focal erythema or red spots in 3, and a few small mucosal erosions or breaks in 2.

Alarm signs were present in 4 of the 62 patients. These included weight loss in a patient with Crohn's disease, anemia in a patient with hookworm, both weight loss and anemia in a patient with middle grade malignant GISTs, and an elevated ESR in a patient with tuberculous peritonitis. All 4 patients who had alarm signs had clinically significant findings (100%, 95% CI = 40–100%). In contrast, of the 58 patients without alarm signs, only 5 had clinically significant findings (8.6%, 95% CI = 2.9–19.0%). This difference between the 2 groups was statistically significant (Fisher's exact test, *P* = .000). 

Of the 9 patients with clinically significant findings on capsule endoscopy, 5 patients (55.6%) had none of the alarm signs defined by the CEDAP-Plus study (95% CI = 21.2–86.3%). These included 3 patients with Crohn's disease and 2 patients with low-grade malignant GISTs.

## 4. Discussion

The introduction of capsule endoscopy into the field of small-bowel diseases diagnosis made definition of indications mandatory on the basis of published data, with suspected mid-gastrointestinal bleeding being the most suitable indication for the procedure. Published reports have described diagnostic yields in the range of 48–76% with this indication. Other potential indications for capsule endoscopy included suspected Crohn's disease, and polyposis syndromes such as familial adenomatosis or Peutz-Jeghers syndrome. Treatment-refractory celiac disease is also conceivably an indication. However, the benefits of capsule endoscopy during diagnostic work-up for chronic abdominal pain and/or diarrhea, the most common reasons referral to gastroenterologists, have remained unclear or controversial [1]. For example, Bardan et al. [2] investigated the yield of capsule endoscopy in 20 patients who suffered from chronic abdominal pain and was in no case able to make a diagnosis capable of explaining the patient's symptoms. Spada et al. [3] used capsule endoscopy to evaluate a cohort of 16 patients with chronic abdominal pain, and only in 1 case obtained a finding correlated with clinical symptoms (6.3%). A retrospective analysis by Fry et al. [4] reported a slightly higher diagnostic yield (9%, 6/64) using capsule endoscopy in patients with chronic abdominal pain or diarrhea. In this retrospective study, we investigated the diagnostic yield of capsule endoscopy in 62 patients with obscure chronic abdominal pain, and found that there were clinically significant findings in only 14.5% of these patients (9/62). Although this represents a relatively low diagnostic yield, it is higher than that of the previous reports and suggests that capsule endoscopy should at least be considered during the diagnostic work-up for the chronic abdominal pain. 

One way to increase the diagnostic yield of capsule endoscopy in the case of chronic abdominal pain is to preselect patients based on other criteria. In an early retrospective study, Shim et al. [5] analyzed clinical findings in 110 patients and found that capsule endoscopy was helpful, with a diagnostic yield of 17%, in patients whose abdominal pain was accompanied with weight loss or elevated serum markers of inflammation. The results of a similar study showed that the presence of concomitant signs of inflammation, malabsorption, or obstruction were a predictive factor for positive capsule endoscopy findings in patients with abdominal pain, with or without diarrhea [6]. In the only published, prospective, multicenter trial to date, the CEDAP-Plus study [7], May et al. reported that careful selection of patients based on the presence of one or more alarm signs might increase the diagnostic yield of capsule endoscopy to as high as 40%. The results of the present study support these observations. Specifically, whereas all 4 patients who had the alarm signs specified in the CEDAP-Plus Study had clinically significant findings on capsule endoscopy, significant findings were found in only 5 of 58 patients without these alarm signs. 

It is important to note that the absence of alarm signs does not indicate that capsule endoscopy is unlikely to result in clinically significant findings in these patients. In the current study, 5 of the 9 patients with significant endoscopic findings had no history of any of the CEDAP-Plus alarm signs. Thus, if patients had been prescreened, with the presence of alarm signs being an absolute requirement for eligibility for capsule endoscopy, 55% of patients in which this technique was potentially useful would have been missed. These results suggest that the alarm signs defined by the CEDAP-Plus study are of relatively low sensitivity in this group of patients. 

In conclusion, patient selection on the basis of alarm signs may increase the yield of capsule endoscopy in patients with obscure chronic abdominal pain. However, as the alarm-sign criteria defied by the CEDAP-Plus Study appear to be of low sensitivity, future research should concentrate on uncovering new and more sensitive screening criteria.
